# Nonlinear Associations between Blood Cadmium Concentration and Thyroid Hormones According to Smoking Status in Korean Adults: The Korea National Health and Nutrition Examination Survey (KNHANES)

**DOI:** 10.3390/toxics11020129

**Published:** 2023-01-29

**Authors:** Byungmi Kim, Minshik Rhie, Sunhee Park, Hyo-Seon Kim, Jeoung A Kwon

**Affiliations:** 1 Division of Cancer Prevention, National Cancer Control Institute, National Cancer Center, Goyang 10408, Republic of Korea; 2 Center of Tobacco Control, National Cancer Center, Goyang 10408, Republic of Korea; 3 Public Health at Graduate School, The Catholic University of Korea, Seoul 06591, Republic of Korea; 4 Institute of Health Services Research, Yonsei University, Seoul 03722, Republic of Korea

**Keywords:** blood cadmium, thyroid hormones, smoking status, restricted cubic splines

## Abstract

Research on the association between blood cadmium (BCd) exposure and thyroid hormone levels in the general population has been inconclusive. Therefore, we examined the associations between BCd and thyroid hormones according to smoking status in Korean adults (N = 1170, Men = 722, Women = 448) using multiple linear regression and restricted cubic splines analysis with data from the Korean National Health and Nutrition Examination Survey (2013). The geometric mean of BCd was 0.74 μg/L in all study participants and was higher in smokers (1.01 μg/L) than in nonsmokers (0.65 μg/L). Restricted cubic splines analysis revealed nonlinear trends between BCd and free thyroxine in smokers (*p* for nonlinearity = 0.02). By contrast, there were no significant associations between BCd and thyroid hormones in either men or women. In conclusion, nonlinear associations may exist between BCd and free thyroxine in smokers. Our study provides empirical support for the future formulation of an acceptable concentration range of BCd and offers a new concept for preventing thyroid problems.

## 1. Introduction

Cadmium (Cd) is a toxic heavy metal and one of the main endocrine disruptors (ED) that disturbs endogenous hormone action [1,2]. Cadmium is contained in batteries, pigments, coatings, and crops and can travel through soil, water, and air. For the general population, cigarette smoking is a major source of cadmium exposure [3,4]. In nonsmokers, foods such as lettuce, spinach, potatoes, peanuts, soybeans, and sunflower seeds are the most important source of cadmium exposure [4,5].

The toxicity of Cd has cancer-causing effects [6], and affects kidney function [7], the cardiovascular system [8], diabetes [9], reproductive function [10], bone health [11], and mortality [12]. Also, the thyroid gland is a possible site of high Cd concentration in the body because of cysteine-rich proteins, known as metallothioneins (MT) [13]. Thyroid hormones affected by Cd include the thyroid-stimulating hormone (TSH), thyroid peroxidase antibody (TPOAb), and free thyroxine (fT4), which are the most conventional indicators of thyroid dysfunction, including hypothyroidism, hyperthyroidism, and autoimmune thyroid diseases [2]. However, the results of these studies are still controversial. Increased levels of cadmium may result in elevated, decreased, or unchanged triiodothyronine (T3), thyroxine (T4), TSH, or thyroid autoantibodies [2,14]. 

In previous studies, blood cadmium (BCd) values were 3.5 times higher in smokers’ blood compared to that of nonsmokers in Serbia [4]. BCd was 7.7 times higher in current smokers than in nonsmokers, and 1.5 times higher in former smokers than in nonsmokers [15]. The Substances and Disease Registry [16] showed smoking 20 cigarettes per day deposits 2–4 μg of small, suspended Cd particles into the lungs, and 50–100% of deposited Cd in the alveoli will be absorbed [3,4,17,18,19]. 

Many studies have examined the relationship between BCd and thyroid hormones [2,14,20,21,22], although the findings have been inconsistent. In addition, investigations into the potential effects of BCd on thyroid hormones that consider smoking status and dose–response associations are rare. In this study, we hypothesized that the relationship between BCd and thyroid hormones according to smoking status is nonlinear, and examined this relationship in South Korean adults in detail using data from the Korea National Health and Nutrition Examination Survey (KNHANES).

## 2. Materials and Methods

### 2.1. Data Source and Study Population

**Figure 1:**
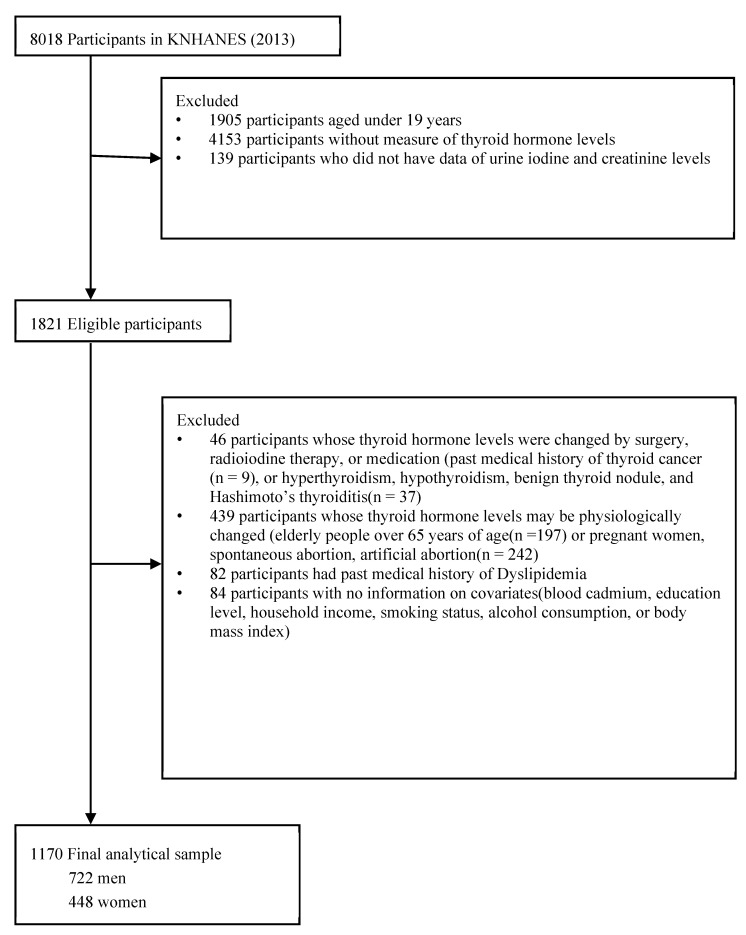
Flow diagram showing study sample derivation. Abbreviations: KNHANES, Korean National Health and Nutrition Examination Survey.

This cross-sectional study was based on data from the Korea National Health and Nutrition Examination Survey (KNHANES), 2013. KNHANES is a cross-sectional, population-based, nationwide survey that is regularly conducted by the Korea Centers for Disease Control and Prevention. Detailed information on the data collection design and process of KNHANES has been published previously [23]. Of the 8018 participants, we excluded those with age under 19 years old (n = 1905), missing thyroid hormone measurements (n = 4153), or missing urine iodine and creatinine levels (n = 139). The blood tests for heavy metals were performed on a randomly selected one-third of the participants aged ≥10 years, by region, sex, and age [23]. The exclusion criteria were as follows: (1) participants whose thyroid hormone levels were altered by surgery, radioiodine therapy, or medication (i.e., a past medical history of thyroid cancer (n = 9) or hyperthyroidism, hypothyroidism, benign thyroid nodule, and Hashimoto’s thyroiditis (n = 37)); (2) participants whose thyroid hormone levels may be physiologically changed (people over 65 years of age (n = 197) or pregnant women, women who have had a spontaneous abortion, or women who have had an artificial abortion (n = 242); (3) participants had past medication history of Dyslipidemia (n = 82); and (4) participants with no covariate information (education level, household income, smoking status, alcohol consumption, or body mass index) (n = 84). We included 1170 participants (722 men and 448 women) in this study (Figure 1).

### 2.2. Cd Concentration Measurements in the Whole Blood

To determine the concentrations of BCd (μg/L) in venous whole blood, blood samples (3 mL) were collected from each participant into blood collection tubes containing ethylenediaminetetraacetic acid (EDTA) for determination of trace elements (BD Vacutainer K2-EDTA tubes; Becton Dickinson, Franklin Lakes, NJ, USA). BCd was measured by graphite furnace atomic absorption spectrometry using the model AAnalyst 600 (Perkin Elmer, Turku, Finland).

Analyses of all blood metals were conducted by the Neodin Medical Institute, a laboratory certified by the Korea Ministry of Health and Welfare. Internal quality assurance and control were established using commercial reference materials (Lyphochek Whole Blood Metals Control; Bio-Rad Laboratories, Hercules, CA, USA). For external quality assurance and control, the Neodin Medical Institute fulfilled the requirements of both the German External Quality Assessment Scheme (operated by Friedrich-Alexander University) and the Quality Assurance Program (operated by the Korea Occupational Safety and Health Agency). The institute was also certified by the Ministry of Labor as a designated laboratory for the analysis of specific chemicals, including heavy metals. The method detection limit for Cd was 0.016 μg/L. All samples were above this detection limit.

### 2.3. Outcome Assessment

We collected approximately 15 mL of blood for the analysis of serum TSH, fT4, and TPOAb levels. Within 30 min after separation of the serum, the sample was transferred to the testing facility. The specimens were analyzed by an electrochemiluminescence immunoassay within 24 h. Serum TSH, fT4, and TPOAb levels were measured with an electrochemiluminescence immunoassay method with a Cobas8000 E-602 (Roche Diagnostics, Mannheim, Germany). The reported results of TSH, fT4, and TPOAb measurements met the specifications regarding the accuracy, general chemistry, special immunology, and ligand measurements of the quality control and quality assurance program of the College of American Pathologists [24]. Hormone levels were not normally distributed and were ln-transformed before analysis. UI concentrations were measured with an inductively coupled plasma mass spectrometry device (ICP-MS; Perkin Elmer ICP-MS, Waltham, MA, USA). UI concentrations were measured using an iodine standard (Inorganic Venture, Christiansburg, VA, USA). Iodine concentrations were adjusted using creatinine concentrations to correct for variable water excretion rates at the time of spot urine specimen collection. 

### 2.4. Covariates

We considered the following as covariates: age, sex, educational level, monthly household income, marital status, BMI, smoking status, alcohol consumption, the urine iodine-to-creatinine ratio (UI/Cre), and other heavy metal (Pb and Hg) levels in the blood. Information on education level, monthly household income, marital status, smoking status, and alcohol consumption was collected through a health interview. Educational level was classified as: less than elementary school, middle school, high school, and college or more. Monthly family income was calculated using standardized classification by sex, residence, and five-year age groups and compared with the standard income level of Korean citizens. Monthly family income was then classified into quartiles.

Smoking status was classified as never or ever-smoker (former or current). A never smoker was defined as a person who reported never having smoked 100 cigarettes. Pack-years were calculated by multiplying the number of packs of cigarettes smoked per day by the number of years the person has smoked (one pack has 20 cigarettes). Alcohol consumption was classified as never or ever-drinker (moderate or heavy). BMI was calculated as the participant’s weight in kilograms divided by their height in meters squared. BMI cut-point categories were recommended by World Health Organization (WHO) experts in Asian populations as follows: <18.5 kg/m^2^ for underweight, 18.5–23 kg/m^2^ for normal weight, 23–27.5 kg/m^2^ for overweight, and ≥27.5 kg/m^2^ for obese [25]. 

### 2.5. Statistical Analysis

We examined differences in the following characteristics between men and women: age, educational level, monthly household income, marital status, smoking status, BMI, alcohol consumption, other heavy metal (Pb and Hg) levels in the blood, and the urine iodine-to-creatinine ratio (UI/Cre). 

The geometric means and 95% confidence intervals (CIs) of BCd concentrations were calculated according to age group, educational level, monthly household income, marital status, smoking status, BMI, and alcohol consumption. We further calculated the means and CIs of thyroid function according to sex.

BCd concentrations were natural-log-transformed due to their right-skewed distribution. We performed crude and adjusted multiple linear regression analyses for TSH, fT4, and TPOAb levels in terms of natural-log-transformed BCd concentrations. We also applied five-knot restricted cubic spline analysis (with knots at the 10th, 25th, 50th, 75th, and 90th percentiles) to evaluate the nonlinear, inverted U-shaped associations between natural-log-transformed BCd concentrations and TSH, fT4, and TPOAb levels estimated by the adjusted multiple linear regression analyses. Likelihood ratio tests were performed to compare the linear terms with the cubic spline terms to test for nonlinearity. In order to determine the appropriateness of a linear or non-linear relationship between a dependent variable and independent variables, likelihood ratio tests (LRT) were performed.

Differences in basic characteristics between the two groups (men vs. women) were explored using the PROC SURVEYFREQ procedure (Rao-Scott Chi-square test) for categorical variables and the PROC SURVEYREG procedure (Independent *t*-test) for continuous variables. PROC SURVEYREG analyses with and without covariates were conducted to evaluate the relationship between BCd and thyroid function. There statistical analyses accounted for the complex sampling design. SAS software, version 9.4 (SAS Institute, Cary, NC, USA), was used for data analysis. We carried out the restricted cubic splines analysis for graphical displays using the ‘rms’ package (Frank E Harrell Jr (2022), rms: Regression Modeling Strategies, R package version 6.3-0, (https://CRAN.R-project.org/package=rms (accessed on 1 January 2023). All statistical analyses were two-sided with a significance level of *p* < 0.05.

## 3. Results

The baseline characteristics of the study participants according to sex are presented in Table 1. There were 722 (61.71%) men and 448 (38.29%) women. The mean age of women was higher than in men (*p* < 0.001). Men were significantly more likely to be current smokers and to consume alcohol compared with women. The lower BMI group (<23 kg/m^2^) had a higher proportion of women than men, and the proportion of men was greater than that of women in the higher BMI group (≥23 kg/m^2^).

Table 2 shows BCd concentrations and thyroid hormones in the study population. The geometric mean of BCd concentrations in the entire study sample and in men and women were 0.744, 0.717, and 0.792 μg/L, respectively. We observed gender differences in thyroid function. Women had higher levels of TSH and TPOAb than men (TSH: 2.377 μI U/mL in men vs. 2.888 μI U/mL in women; TPOAb: 9.626 μI U/mL in men vs. 14.163 μI U/mL in women), and men had higher levels of fT4 than women (1.277 μg/dL in men vs. 1.171 μg/dL in women).

Table 3 shows the BCd concentrations according to the characteristics of the study population. For the overall population, men, and women, BCd concentration varied significantly according to age, education level, marital status, and smoking status (*p* < 0.05). Smokers had significantly higher mean BCd concentrations than nonsmokers. However, BCd concentration did not significantly differ according to household income in women, BMI in men and women, and alcohol consumption in the overall population, men, and women.

Table 4 shows the association between BCd concentrations and thyroid hormones according to smoking status. Model 1, a crude model without adjustments, revealed significant results for fT4 in total (*β* = −0.039, *p* < 0.0001), TSH (*β* = 0.289, *p* = 0.027), and fT4 (*β* = −0.063, *p* < 0.0001) in non-smoking group. After adjusting for covariates, there were no significant results.

**Figure 2:**
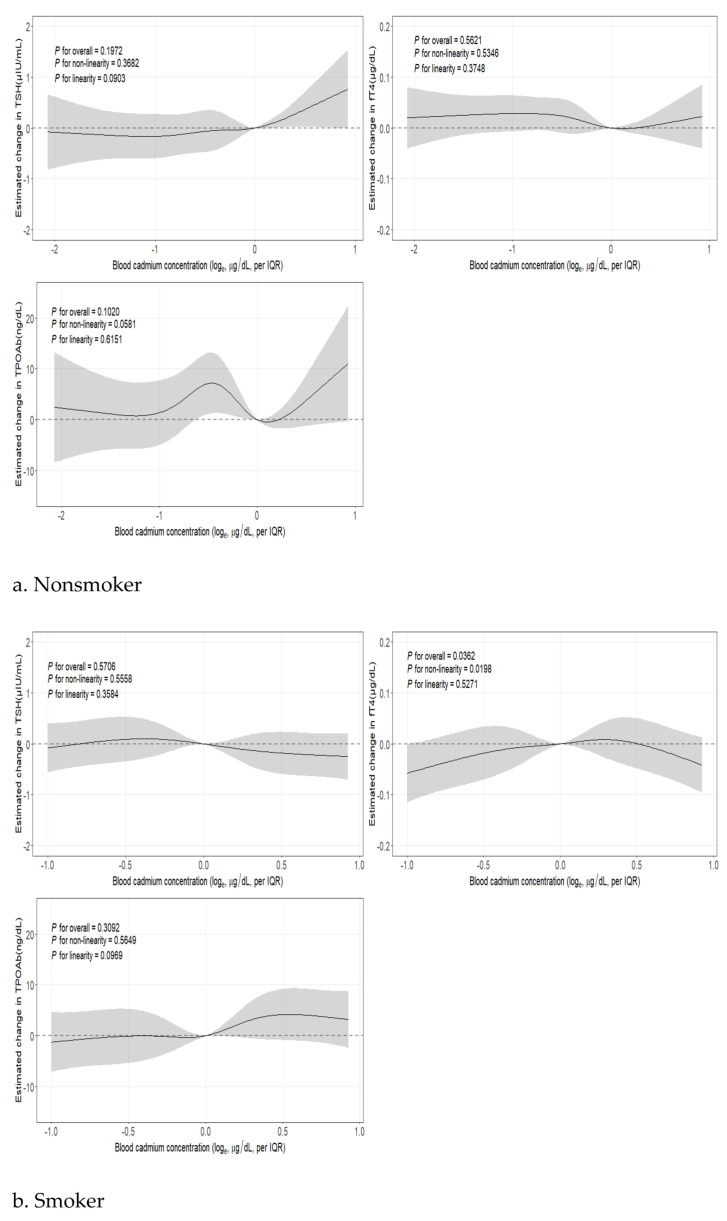
Nonlinear association between BCd and thyroid hormones (TSH, fT4, TPOAb) by smoking status ^a,b^. ^a^ Adjusted for age, sex, education level, smoking status, marital status, household income, body mass index, alcohol consumption, blood lead, blood mercury, and urine iodine-to-creatinine ratio. ^b^ The solid lines show the fitted five-knot spline relationship using a referent 50th percentile of blood lead; the shaded parts indicate the 95% CIs.

In addition, the restricted cubic splines (Figure 2) show the nonlinear association between BCd and thyroid hormones according to smoking status in the adjusted model. There were significant nonlinear associations between BCd concentrations and fT4 levels in a group of smokers. These nonlinear associations were not significant in other groups.

## 4. Discussion

Endocrine disruptors (ED) are defined as chemicals that disrupt hormone function. Among several chemical substances, Cd is representative. Thyroid hormone is among the many hormones affected by exposure to Cd. The reason for the high accumulation of Cd in the thyroid is that metallothioneins (MT), which are proteins rich in cysteine, exist in the thyroid, and Cd binds well to molecules rich in sulfhydryl groups such as MT or glutathione. When humans are exposed to Cd, it can become toxic to the endocrine system, resulting in problems such as hyperthyroidism or hypothyroidism. As such, thyroid toxicity can be confirmed by glandular synthesis, secretion, disease, etc. above T4, T3, and TSH levels. Also, one of the causes of exposure to Cd is smoking. That is why it is so important to look at the difference between smokers and nonsmokers [2]. Therefore, this study investigated the association between BCd and thyroid hormones according to smoking status in South Korean adults using KNHANES data. We found that BCd and fT4 were negatively related among all subjects and in the nonsmoking group in Model 1. Moreover, BCd and TSH were positively related in the nonsmoking group in Model 1. Model 2 produced no significant results. We also investigated whether there are nonlinear associations between BCd and thyroid hormones by smoking status and found one such association between BCd and fT4 in a group of smokers. 

Previous studies on the relationship between Cd and thyroid hormones reported conflicting results. Thyroid-related hormones such as T3, T4, and TSH and thyroid antibodies could be increased, decreased, or not changed by increasing blood or urinary Cd level [2]. The U.S. [20,21,22,26], Korea [14], and China [27] conducted nationwide studies regarding the relationship between Cd and thyroid hormones. In the U.S., Chen et al. [26] found a positive relationship between urinary Cd and total T4 (TT4), total T3 (TT3), and free T3. Christensen et al. showed a negative relationship between BCd and TSH and a positive relationship between urinary Cd and both T3 and T4 [20]. Luo and Hendryx et al. demonstrated a positive relationship between BCd and fT4 and log-thyroglobulin [21], a positive relationship between Cd and total T3 and log-thyroglobulin in men, and a positive relationship between Cd and log-thyroglobulin in women. In Korea, Chung et al. mentioned a negative relationship between BCd and fT4 in total, a negative relationship between BCd and fT4 levels in men, and a positive relationship between BCd and hypothyroidism in men [14]. In China, Chen et al. found a positive relationship between natural log (ln) BCd and ln TGAb (thyroglobulin antibodies) in women [26].

These previous studies focused on either urinary Cd or BCd levels [20,21,22,26]. Our study is based on BCd level. There is a difference in urinary Cd and BCd. Urinary Cd represents the kidney Cd level, which is the cumulative Cd level and represents the body burden. BCd indicates the recent Cd level [17]. Also, the effect of sex on the relationship between Cd and thyroid hormones is important and could be explained by the degree of dysregulation in the pituitary-thyroid axis, toxicokinetics, and hormonal differences by sex [2,21]. Therefore, we conducted a sex-specific analysis on the relationship between Cd and thyroid hormones. Also, the relationship between BCd and thyroid hormone by smoking status was considered. Smoking is one of the main sources of Cd [28]. One serving of tobacco has 1–3 μg Cd [29,30,31], and smokers take in 10% of their Cd intake from tobacco [12]. Therefore, we specified smoking status in the analysis of the relationship between BCd and thyroid hormones.

Our study found significant relationships between BCd and thyroid hormones in Model 1 before adjustment (fT4 in the overall and nonsmoking groups and TSH in the nonsmoking group), but Model 2, which adjusted for various variables, showed no significant associations. Similar results were obtained when the groups were analyzed separately by sex (Supplementary Material, Table S1). However, we assessed nonlinearity to obtain more reliable results, and the analysis according to smoking status showed a significant nonlinear relationship between BCd and fT4 in smokers. Our study is meaningful because no previous studies that analyzed the relationships between BCd and thyroid hormones confirmed the nonlinearity of relationships by sex or smoking status.

This study has some limitations. We used cross-sectional data, which could not explain the casual relationship. Also, we only included BCd levels, which indicate the recent Cd level. We could not consider indirect smoking status in this study. However, despite these limitations, our study is the first to consider the nonlinearity in the relationship between BCd and thyroid hormones by smoking status. Also, fT4 was measured, which is a more effective means of assessing thyroid dysfunction compared to T3 and T4 levels [14].

## 5. Conclusions

This study investigated the associations between BCd and thyroid hormones according to smoking status in Korean adults. Examining the nonlinear associations revealed significant results for BCd and fT4 in smokers. Further prospective studies are required to confirm the nonlinear associations between BCd and thyroid hormones. Our study provides empirical support for the future formulation of an acceptable concentration range of BCd and provides evidence of the need for strategies to reduce exposure to BCd.

## Figures and Tables

**Table 1:** Characteristics of study population in KNHANES study (n = 1170).

Characteristic	N (%)			*p*-Value
	Total	Men 722 (61.71%)	Women 448 (38.29%)	
**Age (years)**				
19~39	651 (53.81)	360 (49.58)	291 (61.04)	<0.001
40~59	519 (46.19)	362 (50.42)	157 (38.96)	
**Education level**				
Elementary school or less	59 (4.570)	44 (5.70)	15 (2.63)	0.011
Middle school	79 (7.54)	57 (8.63)	22 (5.68)	
High school	520 (44.67)	325 (45.15)	195 (43.86)	
College or more	512 (43.22)	296 (40.53)	216 (47.83)	
**House income**				
Low	103 (8.26)	74 (9.58)	29 (6.01)	0.092
Middle	649 (56.51)	388 (55.25)	261 (58.66)	
High	418 (35.22)	260 (35.17)	158 (35.33)	
**Marital status**				
Married	713 (62.30)	429 (60.60)	284 (65.19)	0.346
Unmarried	400 (33.73)	255 (35.33)	145 (31.01)	
Divorced/Widowed/separated	53 (3.97)	34 (4.07)	19 (3.80)	
**Body mass index (kg/m^2^)**				
<23.0	543 (44.83)	269 (36.91)	274 (58.36)	<0.001
≥23.0	627 (55.17)	453 (63.09)	174 (41.64)	
**Smoking status**				
Nonsmoker	813(69.02)	399(54.96)	414(93.06)	<0.001
Smoker	354(30.98)	321(45.04)	33(6.94)	<0.001
**Alcohol consumption**				
Nondrinker	362 (32.13)	160 (23.39)	202 (47.1)	<0.001
Drinker	806 (67.87)	561 (76.61)	245 (52.9)	
**UI/Cre (ug/g), GM(SE)**	2.07 ± 0.08	1.88 ± 0.09	2.44 ± 0.17	0.047
**BPb (μg/dL), GM(SE)**	1.90 ± 0.03	2.17 ± 0.04	1.51 ± 0.03	<0.001
**BHg (μg/L), GM(SE)**	3.27 ± 0.07	3.67 ± 0.09	2.68 ± 0.08	<0.001

Abbreviations: KNHANES, Korean National Health and Nutrition Examination Survey; UI/Cre, urine iodine-to-creatinine ratio; GM(SE): Geometric Mean (Standard error.); BPb, blood lead; BHg, blood mercury.

**Table 2:** Distribution of BCd concentrations (μg/L) and thyroid hormones in the study population.

	Total	Men	Women	*p*-Value
**BCd Concentrations GM (GSD)**	0.744 (2.313)	0.717 (1.255)	0.792 (8.035)	0.100
Percentile				
25 th	0.501	0.472	0.540	
50 th	0.789	0.768	0.829	
75 th	1.160	1.112	1.190	
**Thyroid hormones (Mean/SD)**				
TSH (μIU/mL)	2.573 (1.868)	2.377 (1.543)	2.888 (2.263)	<0.001
fT4 (μg/dL)	1.236 (0.175)	1.277 (0.173)	1.171 (0.159)	<0.001
TPOAb (ng/dL)	11.363 (26.125)	9.626 (18.658)	14.163 (34.795)	0.031

Abbreviations: BCd, blood cadmium; GM(SE): Geometric Mean (Standard error.). TSH, Thyroid-stimulating hormone; fT4, free thyroxin; TPOAb, anti-thyroid peroxidase antibody.

**Table 3:** BCd Concentrations(μg/L) according to characteristics of study population.

Characteristics	BCd Concentration (μg/L)					
	Total		Men		Women	
	GSMean (GSD)	*p*-Value	GSMean (GSD)	*p*-Value	GSMean (GSD)	*p*-Value
**Age (years)**						
19~39	0.578 (6.395)	<0.001	0.546 (1.770)	<0.001	0.626 (1.340)	<0.001
40~59	0.997 (5.460)		0.936 (2.468)		1.146 (7.935)	
**Education level**						
Elementary school or less	0.987 (4.526)	<0.001	0.936 (2.623)	<0.001	1.200 (1.002)	0.007
Middle school	1.048 (1.240)		1.037 (2.457)		1.077 (4.061)	
High school	0.735 (3.181)		0.684 (2.160)		0.833 (2.040)	
College or more	0.688 (8.859)		0.671 (5.259)		0.713 (3.481)	
**Household income**						
Low	0.902 (5.655)	0.008	0.856 (1.846)	0.013	1.043 (2.332)	0.093
Middle	0.749 (8.013)		0.740 (4.788)		0.765 (5.854)	
High	0.702 (1.694)		0.649 (1.005)		0.802 (5.930)	
**Marital status**						
Married	0.874 (1.184)	<0.001	0.846 (5.990)	<0.001	0.921 (2.196)	<0.001
Unmarried	0.523 (7.657)		0.514 (4.958)		0.540 (3.252)	
Divorced/Widowed/Separated	1.215 (1.793)		1.137 (2.385)		1.372 (2.112)	
**Body mass index (kg/m^2^)**						
<23.0	0.739 (2.472)	<0.001	0.717 (2.220)	0.304	0.763 (1.209)	0.112
≥23.0	0.748 (2.336)		0.716 (1.241)		0.835 (7.418)	
**Smoking status**						
Nonsmoker	0.647 (1.987)	<0.001	0.540 (1.816)	<0.001	0.777 (2.083)	0.028
Smoker	1.008 (5.013)		1.009 (3.296)		1.004 (1.591)	
**Alcohol consumption**						
Nondrinker	0.762 (7.907)	0.961	0.721 (1.910)	0.648	0.799 (2.841)	0.971
Drinker	0.734 (1.050)		0.715 (4.398)		0.784 (2.377)	

Abbreviations: BCd, blood cadmium; TSH, thyroid-stimulating hormone; fT4, free thyroxine; TPOAb, anti-thyroid peroxidase antibody; UI/Cre, urine iodine-to-creatinine ratio.

**Table 4:** Associations between the Cd Concentration (log Transformed) and thyroid hormones.

Outcome	Total		Non-Smoking		Smoking	
	*β* (SE)	*p*-Value	*β* (SE)	*p*-Value	*β* (SE)	*p*-Value
Model 1 ^a^						
TSH	0.017 (0.092)	0.854	0.289 (0.13)	0.027	−0.224 (0.135)	0.099
fT4	−0.039 (0.009)	<0.0001	−0.063 (0.011)	<0.001	−0.034 (0.019)	0.066
TPOAb	1.567 (1.074)	0.146	2.789 (1.587)	0.081	1.605 (0.908)	0.079
Model 2 ^b^						
TSH	0.098 (0.112)	0.384	0.175 (0.139)	0.209	−0.120 (0.121)	0.322
fT4	−0.008 (0.009)	0.374	−0.010 (0.011)	0.389	0.000 (0.017)	0.977
TPOAb	0.708 (1.471)	0.631	−0.273 (2.037)	0.894	1.753 (1.224)	0.154

Abbreviations: TSH, thyroid-stimulating hormone; fT4, free thyroxine; TPOAb, anti-thyroid peroxidase antibody. Adjusted for age, sex, education level, marital status, houshold income, body mass index, alcohol consumption, blood lead, blood mercury, and urine iodine-to-creatinine ratio. ^a^ Model 1: crude model without adjustment. ^b^ Model 2: adjusted for age, education level, marital status, household income, body mass index, smoking status, alcohol consumption, blood lead, blood mercury, and urine iodine to-creatinine ratio

## Data Availability

Publicly available datasets were analyzed in this study. These data can be found at https://www.kdca.go.kr/yhs/ (accessed on 1 December 2022).

## Supplementary Materials

The following supporting information can be downloaded at https://www.mdpi.com/article/10.3390/toxics11020129/s1: Table S1: Associations between the Cd Concentration (log Transformed) and thyroid hormones; Figure S1: Non-linear association between BCd and thyroid hormones (TSH, fT4, TPOAb) by sex.

## Abbreviations

Cd, cadmium; TSH, thyroid-stimulating hormone; TPOAb, thyroid peroxidase antibody; fT4, free thyroxine; BCd, blood cadmium; WHO, The World Health Organization; CDC, Centers for Disease Control and Prevention; BMI, body mass index; KNHANES, Korea National Health and Nutrition Examination Survey; EDTA, ethylenediaminetetraacetic acid; ICP-MS, inductively coupled plasma mass spectrometry; Pb and Hg, lead and mercury; UI/Cre, urine iodine-to-creatinine; ED, endocrine disruptors; MT, metallothioneins; TT4, total T4; TT3, total T3; TGAb, thyroglobulin antibodies.
