# Supplementary material for: Nonlinear Associations between Blood Cadmium Concentration and Thyroid Hormones According to Smoking Status in Korean Adults: The Korea National Health and Nutrition Examination Survey (KNHANES)
Source: Toxics. 2023 Jan 29;11(2):129. doi: 10.3390/toxics11020129 (PMC9958680; doi:10.3390/toxics11020129)
Supplement: Supplementary file 1 [file toxics-11-00129-s001.zip › toxics-2182952-supplementary.pdf]

Supplementary Information

Table S1. Associations between the Cd Concentration (log Transformed) and thyroid hormones.

Outcome	Total		Men		Women	
	**β (SE)**	***p*-Value**	**β (SE)**	***p*-Value**	**β (SE)**	***p*-Value**
**Model 1[a]**						
TSH	0.017(0.092)	0.854	−0.208(0.075)	0.006	0.332(0.220)	0.132
fT4	−0.039(0.009)	<.0001	−0.034(0.012)	0.004	−0.032(0.011)	0.003
TPOAb	1.567(1.074)	0.146	0.612(0.803)	0.447	2.621(2.566)	0.309
**Model 2[b]**						
TSH	0.098(0.112)	0.384	−0.051(0.100)	0.608	0.341(0.223)	0.129
fT4	−0.008(0.009)	0.374	−0.005(0.014)	0.709	−0.018(0.012)	0.123
TPOAb	0.708(1.471)	0.631	1.155(0.829)	0.165	−0.743(3.896)	0.849

Abbreviations : TSH, thyroid-stimulating hormone ; fT4, free thyroxine; TPOAb, anti-thyroid peroxidase antibody. Adjusted for age, education level, smoke status, marital status, household income, Body mass index, alcohol consumption, blood lead, blood mercury, and urine iodine to-creatinine ratio

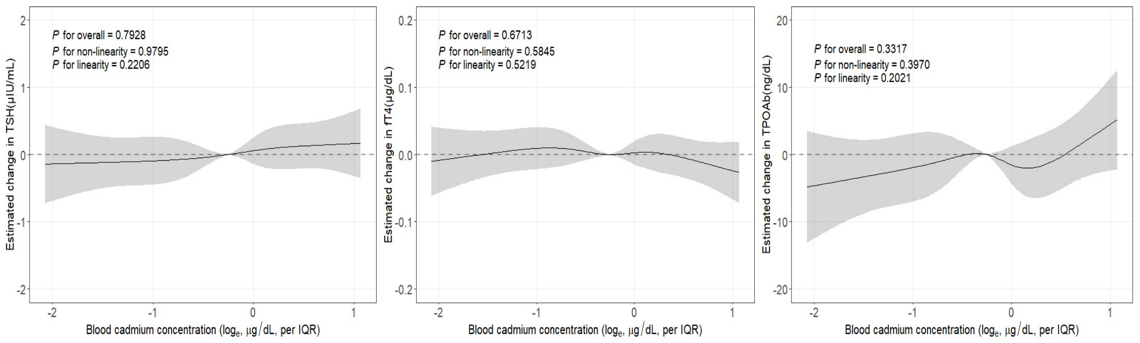

A. Total [b]

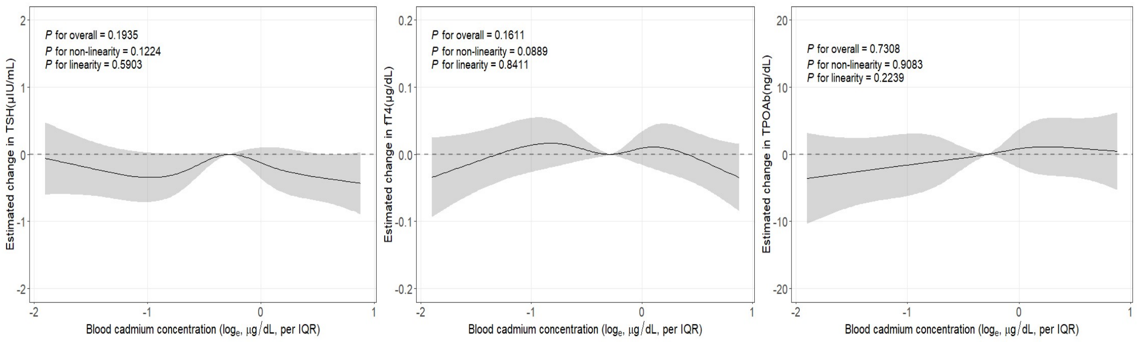

B. Men [a]

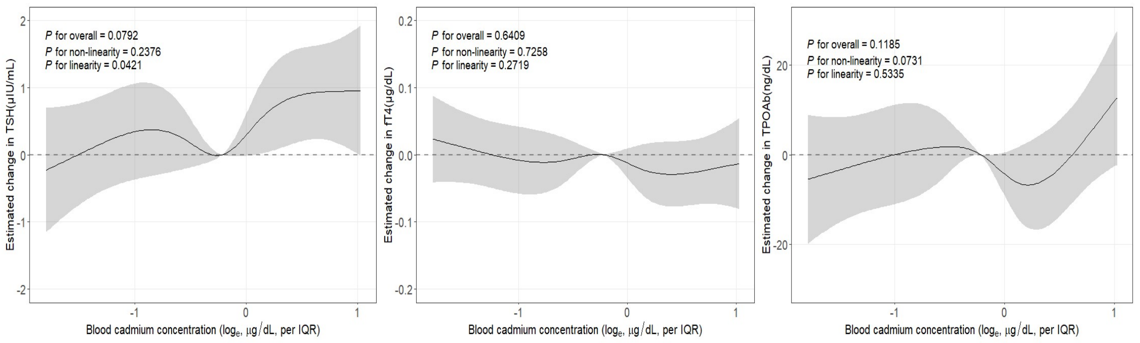

C. Women [a]

Figure S1. Non-linear association between BCd and thyroid hormones (TSH, fT4, TPOAb) by sex [c]. [(a)] Model adjusted for age, sex, education level, smoking status, marital status, household income, body mass index, alcohol consumption, blood lead, blood mercury, and urine iodine-to-creatinine ratio. [(b)] Adjusted for variables in model plus sex in total. [(c)] The solid lines show the fitted five-knot spline relationship using a referent 50th percentile of blood lead; the shaded parts indicate the 95% CIs.
